# Single-Cell Multi-Omics: Insights into Therapeutic Innovations to Advance Treatment in Cancer

**DOI:** 10.3390/ijms26062447

**Published:** 2025-03-09

**Authors:** Angel Guan, Camelia Quek

**Affiliations:** 1 Melanoma Institute Australia, The University of Sydney, Sydney, NSW 2065, Australia; agua7813@uni.sydney.edu.au; 2 Faculty of Medicine and Health, The University of Sydney, Sydney, NSW 2006, Australia; 3 Charles Perkins Centre, The University of Sydney, Sydney, NSW 2006, Australia

**Keywords:** multi-omics, single-cell, sequencing, tumour microenvironment, cancer treatment, translational research, resistance

## Abstract

Advances in single-cell multi-omics technologies have deepened our understanding of cancer biology by integrating genomic, transcriptomic, epigenomic, and proteomic data at single-cell resolution. These single-cell multi-omics technologies provide unprecedented insights into tumour heterogeneity, tumour microenvironment, and mechanisms of therapeutic resistance, enabling the development of precision medicine strategies. The emerging field of single-cell multi-omics in genomic medicine has improved patient outcomes. However, most clinical applications still depend on bulk genomic approaches, which fail to directly capture the genomic variations driving cellular heterogeneity. In this review, we explore the common single-cell multi-omics platforms and discuss key analytical steps for data integration. Furthermore, we highlight emerging knowledge in therapeutic resistance and immune evasion, and the potential of new therapeutic innovations informed by single-cell multi-omics. Finally, we discuss the future directions of the application of single-cell multi-omics technologies. By bridging the gap between technological advancements and clinical implementation, this review provides a roadmap for leveraging single-cell multi-omics to improve cancer treatment and patient outcomes.

## 1. Introduction

In cancer, malignant cells do not function in isolation but exist within a complex tumour microenvironment (TME) composed of diverse stromal, immune, and endothelial cell populations that influence tumour progression and therapeutic response [1]. Each cell in TME exists a complex, interactive hierarchy of molecular layers—from genome and epigenome to transcriptome, proteome, and metabolome. These layers function together to support cellular processes like proliferation, survival, and metabolism. The TME modulates cell function through receptor–ligand binding, the release of growth factors and cytokines, and environmental conditions such as oxygen level. Understanding interactions between cytotoxic T cells and tumour cells has led to important therapeutic advances. For example, immune checkpoint inhibitors (ICIs) have revolutionised cancer treatment by re-stimulating the immune response, enabling durable response and prolonged disease-free survival for some patients [2]. However, many patients still suffer from innate or acquired resistance, meaning that they either do not respond to ICIs (innate resistance) or initially respond but subsequently relapse (acquired resistance). The complex TME described above is widely verified to be one of the key drivers of resistance and recurrence [3]. Complex biological processes in the TME—including immunosuppressive signalling, metabolic reprogramming, altered angiogenesis, extracellular matrix remodelling, and dynamic crosstalk between cancer and stromal cells—drive tumour growth, progression, immune evasion, and metastasis while adapting to resist therapeutic interventions [4].

Understanding both intrinsic and extrinsic TME complexities is essential for gathering a holistic view of cancer and elucidating the resistance heterogeneity. The combined effect of intrinsic and extrinsic factors can be complex to analyse. Studies often simplify the models by including only part of the system. For example, studying single molecular levels (mono-omics) in non-spatial settings (e.g., single-cell RNA-sequencing [scRNA-seq]) has historically improved our understanding of TME. Tirosh et al. were one of the earliest teams who profiled the transcriptome of the melanoma TME, aimed at exploring the distinct genotypic and phenotypic states of melanoma tumours [5]. They performed scRNA-seq on 4645 single cells from 19 melanoma patients and uncovered the diversity of the TME. They identified tumour-associated fibroblasts, endothelial cells, and various immune cells, which was historically not possible with bulk RNA-seq. Two distinct states of malignant cells—*MITF*-high and *AXL*-high—were found to correlate with therapy resistance. Additionally, they showed that tumour-infiltrating lymphocytes (TILs) exhibited varying degrees of exhaustion, and non-exhausted cytotoxic cells were predominantly non-expanded, suggesting a possible immune evasion strategy. While transformative at the time, mono-omics studies like scRNA-seq frequently assumed that transcriptional states observed in vitro reflect in vivo behaviour, lacking a true link between transcriptional changes and functional changes. To avoid over-simplifying a biological system, methodologies that enable simultaneous interrogation of multiple molecular layers have become crucial. These approaches link genetic mutations in the genome to changes in the epigenome, transcriptome, and proteome at both single-cell and spatial resolution. This is especially important in cancer research given the heterogeneity observed in TME within and between individual patients [1].

**Figure 1:**
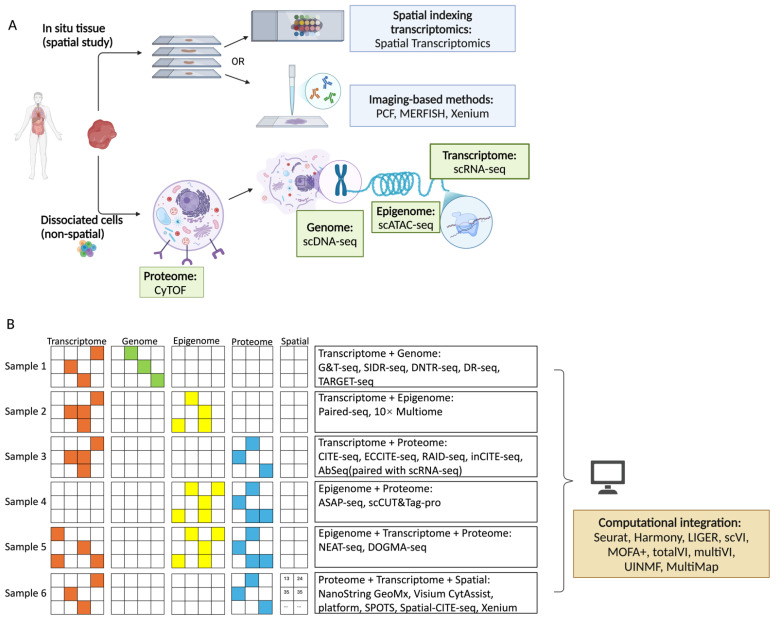
An overview of experimental and computational framework of single-cell multi-omics. (**A**) Selected mono-omics tools: Various mono-omics technologies enable molecular profiling at single-cell or near-single-cell resolution. Spatial transcriptomics methods, such as Spatial Transcriptomics, provide spatially resolved gene expression analysis. Imaging-based techniques, including PCF, MERFISH, and Xenium, offer high-resolution visualisation of molecular markers. CyTOF, scDNA-seq, scATAC-seq, and scRNA-seq profile the proteome, genome, epigenome, and transcriptome, respectively. These technologies form the foundation of single-cell multi-omics. (**B**) Summary of multi-omics and computational integration: Multi-omics methods enable joint profiling of molecular layers, such as transcriptome-genome, transcriptome-epigenome, and proteome-transcriptome. Computational tools, including Seurat, Harmony, LIGER, scVI, MOFA+, totalVI, MultiVI, UNIMF, and MultiMap, integrate these datasets by harmonising data and aligning modalities, aiding the interpretation of complex single-cell multi-omics data. Abbreviations: scRNA-seq—single-cell RNA sequencing, scDNA-seq—single-cell DNA sequencing, scATAC-seq—single-cell Assay for Transposase-Accessible Chromatin sequencing, CyTOF—Cytometry by Time-Of-Flight, PCF—Phenocycler Fusion, MERFISH—Multiplexed Error Robust Fluorescence In Situ Hybridisation, G&T-seq—Genomic and Transcriptomic sequencing, SIDR-seq—Simultaneous DNA and RNA sequencing, DNTR-seq—Direct Nuclear Tagmentation and RNA sequencing, DR-seq—gDNA-mRNA sequencing, CITE-seq—Cellular Indexing of Transcriptomes and Epitopes by sequencing, ECCITE-seq—Expanded CRISPR-compatible Cellular Indexing of Transcriptomes and Epitopes sequencing, RAID-seq—Single-cell RNA and Immunodetection sequencing, inCITE-seq—Intranuclear Cellular Indexing of Transcriptomes and Epitopes sequencing, AbSeq—Oligonucleotide-barcoded Antibodies sequencing, ASAP-seq—ATAC with Select Antigen Profiling by sequencing, NEAT-seq—Sequencing of Nuclear Protein Epitope Abundance, Chromatin Accessibility, and the Transcriptome in Single Cells, SPOTS—Spatial PrOtein and Transcriptome sequencing.

In this review, we introduce single-cell multi-omics assays that allow paired measurements of more than one modality in the same cell. Next, we will discuss how computational tools integrate datasets of the same or different data types. Lastly, we will explore how these single-cell multi-omics technologies have been applied to deepen our understanding in cancer progression and resistance heterogeneity, paving the way for new therapeutic innovations in cancer treatment. In this context, multi-omics studies involve measuring multiple omics either in parallel or sequentially within the same cells or computationally integrating two omics datasets to generate a multi-omics profile (Figure 1). Studies that perform separate mono-omics analyses solely for cross-validation are not the focus of this review.

## 2. Single-Cell Multi-Omics Assays

Single-cell multi-omics assays enable the simultaneous profiling of multiple omics (i.e., DNA, RNA, chromatin, and proteins) from the same cell (Table 1). These assays are broadly divided into single-cell multi-omics and spatial multi-omics. The former typically processes sequencing in dissociated cells, providing greater resolution. The latter preserves the spatial context of cells within tissues, providing insights into how cellular interactions and locations impact biological functions.

### 2.1. Genome and Transcriptome

Simultaneous profiling of the genome and transcriptome in a single cell provides a comprehensive understanding of how genetic alterations influence transcriptomic changes (Figure 1B, sample 1). Integrating both data types enables the detection of phenomena such as gene dosage effects due to copy number variations (CNVs), fusion transcripts resulting from genomic rearrangements, and somatic single nucleotide variants (SNVs) expressed at the transcriptomic level.

The first technology developed to profile DNA and RNA simultaneously was G&T-seq (Genomic and Transcriptomic sequencing) [6]. Macaulay et al. separated RNA and DNA by adding a biotinylated oligo-dT primer, which selectively binds to polyadenylated tail of mRNA. This primer is attached to streptavidin-coated magnetic beads, allowing the captured mRNA to be separated from the cell lysate using a magnet. The isolated RNA is then processed with Smart-seq2 for transcriptome profiling. The DNA is amplified using either PicoPlex (for CNV analysis) or Multiple Displacement Amplification (for whole-genome sequencing [WGS]). Other technologies separate mRNA and genomic DNA (gDNA) via controlled cell lysis. SIDR-seq (Simultaneous DNA and RNA sequencing) selectively lyses the cell membrane while preserving the nuclear envelope, allowing RNA to diffuse out while retaining DNA within the nucleus [7]. Before lysis, cells are incubated with antibody-conjugated magnetic beads, which facilitate the magnetic retention of cell lysates. Once lysis occurs, the RNA-containing supernatant is carefully pipetted out and transferred to a clean tube for further processing. DNTR-seq (Direct Nuclear Tagmentation and RNA sequencing) also uses controlled lysis but separates the nucleus through centrifugation followed by aspiration [8].

Other technologies employ strategies that do not require physical separation. In DR-seq (gDNA-mRNA sequencing), mRNA is selectively reverse transcribed into complementary DNA (cDNA) using a cell-specific primer that contains T7 promoter [9]. The mixture with both cDNA and gDNA is then split: one part undergoes PCR to amplify gDNA, while the other undergoes in vitro transcription to amplify cDNA. While DR-seq reduces the risk of DNA loss compared to physical separation methods, it is susceptible to RNA-derived reads contaminating gDNA analysis. On the other hand, TARGET-seq focuses on generating a mutational profile rather than whole-genome analysis [10]. After single-cell lysis, polyadenylated cDNA and mutations of interest are co-amplified using target-specific primers. The reaction is then split: one for whole-transcriptome library preparation and the other for targeted next-generation sequencing (NGS) of specific cDNA and gDNA amplicons. TARGET-seq is notable for its high sensitivity (98.4%) in detecting both coding and non-coding mutations in a single cell. Additionally, TARGET-seq addresses the challenge of allelic dropout, which can result in the loss of heterozygous mutation detection. However, TARGET-seq has a major limitation. Target-specific primers must be designed for each mutation of interest, making it time-consuming to analyse a large number of mutations. In the original study, Rodriguez-Meira et al. detected 12 different mutations per single cell and the incorporation of more mutations remains to be explored.

All methods described in this section simultaneously profile the genome and transcriptome. However, they differ in sequencing depth, sample compatibility, cost, time, and whether whole-transcriptome sequencing (WTS) or WGS is performed. For instance, DR-seq has only been reported for use in fresh cells, whereas other methods are compatible with frozen cells. DNTR-seq, SIDR-seq, and DR-seq have relatively lower sequencing depths compared to G&T-seq and TARGET-seq, making them more suitable for CNV rather than SNV analysis. G&T-seq has been shown to achieve the highest genomic coverage (78.3%). DNTR-seq is described by its developers as cost-effective, mainly because it avoids specialised instrumentation and relies on ultra-low-coverage sequencing. DNTR-seq, TARGET-seq, and G&T-seq also limit their profiling to polyadenylated RNA. Because no benchmarking study under controlled conditions is available, these differences should be treated only as a general guide for selecting a method.

### 2.2. Transcriptome and Chromatin Accessibility

Simultaneous profiling of chromatin accessibility and the transcriptome in a single cell helps uncover how chromatin remodelling influences gene expression (Figure 1B, sample 2). Integrating the two data types potentially provides insights into regulatory networks, tumour evolution, and identifying epigenetic and transcriptional drivers of tumour heterogeneity and drug resistance.

Joint profiling of the transcriptome and chromatin accessibility in a single reaction is a common strategy. One example is Paired-seq, which uses a ligation-based combinatorial indexing platform [11]. The process begins with Tn5 tagmentation followed by reverse transcription (RT) across eight wells, with each well receiving a unique barcode linked to the Tn5 transposase adaptor and RT primer. The nuclei are then pooled and redistributed into a 96-well plate, where well-specific DNA barcodes are ligated to the DNA fragments and cDNA molecules. Three rounds of ligation generate 10⁷ unique barcodes, giving each nucleus a distinct identity. After preamplification, the DNA molecules are split into two fractions, each subjected to a restriction enzyme that selectively digests pre-designed sites in either the Tn5 or RT primers. Compared to physical separation strategies, joint profiling methods more readily link the transcriptome with the epigenome. Additionally, joint profiling minimises cell content loss and offers a cleaner, more streamlined protocol. In 10× Multiome, nuclei are encapsulated in Gel Beads-in-Emulsion (GEMs) after tagmentation, with each one containing a unique barcode [12]. When the gel beads dissolve, the barcodes tag both polyadenylated mRNA for cDNA synthesis and transposed DNA fragments for chromatin accessibility profiling. The commercially available 10× Multiome is a more automated method than Paired-seq but requires specialised, high-cost reagents and instrumentation.

When selecting a method, factors such as cost, throughput, sensitivity, scalability, and cell coverage must be considered. For instance, a lower-throughput approach (processing thousands to tens of thousands of cells) may be more suitable for tissue profiling and disease modelling. In contrast, an ultra-high-throughput protocol (analysing millions of cells) using a combinatorial indexing strategy may be more appropriate for organism-level analysis or developmental biology.

While chromatin accessibility largely reflects the combined regulatory state of a cell, the epigenome can be profiled at other levels such as DNA methylation, histone modifications, and transcription factor (TF) activity. However, profiling these levels is generally more challenging and, therefore, not widely used in cancer research. The first method developed to profile both the DNA methylation and transcriptome, scM&T-seq (parallel single-cell methylation and transcriptome sequencing), combines Smart-seq2 and scBS-seq (bisulfite sequencing) [13]. Since bisulfite treatment denatures and fragments DNA, purification steps are required, leading to DNA loss. Sample loss is further exacerbated by the physical separation required in scM&T-seq. Profiling histone modifications and TF activity is generally limited by the availability of suitable antibodies. Vandereyken et al. provided a more comprehensive review highlighting the feasibility of co-profiling the transcriptome with different levels of epigenetic modifications [14].

### 2.3. Transcriptome and Protein

While RNA transcripts provide insight into gene expression, proteins are essential for understanding cellular function. Proteins perform diverse roles, including structural support, enzymatic activity, and transport. More importantly, proteins, rather than RNAs, serve as the primary targets for drug development. Thus, simultaneously profiling RNA and proteins is important for studying disease models, supporting functional analysis, and facilitating clinical translation (Figure 1B, sample 3).

CITE-seq (cellular indexing of transcriptomes and epitopes by sequencing) is one of the most widely applied methods and is compatible with high-throughput droplet-based single-cell technologies such as 10× Genomics [15]. Before loading onto the single-cell platform, cells are incubated with DNA-barcoded antibodies that bind to surface proteins of interest. These DNA barcodes are unique to each antibody and contain polyadenylated tails to enable capture alongside mRNA. Subsequent processing is like standard scRNA-seq. Cells are loaded into a droplet-based platform for single-cell encapsulation. Upon release of barcoded beads and cell lysis, the DNA barcodes and mRNA are reverse transcribed using poly-T primers with cell-specific barcodes. Building on the CITE-seq framework, several new technologies have expanded its ability to profile additional modalities. For instance, Mimitou et al. introduced ECCITE-seq (Expanded CRISPR-compatible Cellular Indexing of Transcriptomes and Epitopes sequencing) [16]. ECCITE-seq enables not only surface protein and RNA detection but also CRISPR perturbation profiling. Specifically, cells are transduced with lentiviral vectors carrying designed single-guide RNAs (sgRNAs). Each cell will integrate one or more sgRNAs to generate a pool of genetically perturbed cells. ECCITE-seq incorporates a custom RT primer specific to the sgRNA scaffold sequence, ensuring that the sgRNAs are also reverse transcribed into cDNA and later sequenced. By measuring RNA and protein changes alongside gene perturbations, researchers can map downstream molecular pathways affected by specific gene deletions. More recently, inCITE-seq (Intranuclear Cellular Indexing of Transcriptomes and Epitopes sequencing) allows intranuclear protein profiling, and spatial CITE-seq enables spatially resolved single-cell analyses [17,18]. Nonetheless, single-cell proteomic profiling is typically limited by the availability of antibodies, resulting in low-plex detection. Currently, CITE-seq is commercially available through 10× Genomics and BioLegend, allowing the profiling of approximately 200 proteins in a single experiment [18]. 

RAID-seq (Single-cell RNA and Immunodetection sequencing) focuses on profiling intracellular proteins alongside the transcriptome [19]. To enable intracellular protein staining while preserving endogenous mRNAs, cells are fixed, permeabilised, and crosslinked with reversible crosslinking reagents. The optimised preprocessing protocol allows RNA-barcoded antibody conjugates to cross the plasma membrane and stain intracellular proteins without significant mRNA loss. After reversing the crosslinking, the antibodies with RNA barcodes are profiled alongside mRNAs during RT. The original study demonstrated the detection of six intracellular proteins, including two phosphorylated epitopes. In more recent research, a panel of 70 intracellular proteins was reported [20]. However, fixed cells in RAID-seq exhibited a slight reduction in gene detection, potentially limiting the identification of low-expressed genes. Additionally, variability in antibody-barcode conjugation efficiency makes RAID-seq a time-consuming method that requires extensive optimisation during panel design.

Another commercially available multi-omics platform, AbSeq (Oligonucleotide-barcoded Antibodies sequencing), is less commonly used than CITE-seq [21,22]. AbSeq adopted a targeted transcriptomic profiling strategy and has minimal sequencing depth to analyse low-abundance transcripts with comparable sensitivity. While no comprehensive benchmarking has been conducted across all available technologies, key features of these methods should guide researchers in selecting the most suitable tool. The choice of technique depends on the research question and budget. For instance, when studying a well-defined set of genes in a specific cell type (i.e., CD8 T cells), AbSeq is advantageous as it provides better profiling of low-expressed genes. The smaller library size and shallower sequencing depth also make AbSeq a more cost-effective assay. Conversely, when prior knowledge is limited and the goal is to explore novel genes, pathways, or cell types, the unbiased whole-transcriptome approach of CITE-seq is more appropriate.

### 2.4. Chromatin Accessibility and Protein

Simultaneously profiling the epigenome and proteome enables the linking of cellular function to regulatory states (Figure 1B, sample 4). The most commonly used method, ASAP-seq (ATAC with Select Antigen Profiling by sequencing), utilises oligo-conjugated antibodies to label surface proteins and employs the ATAC-seq workflow to map accessible chromatin regions [23]. Both data types are captured within the same droplet-based system, enabling multimodal analysis of protein expression and epigenetic regulation in individual cells. Compared to RNA-based methods like CITE-seq, detecting surface proteins alongside chromatin accessibility is challenging because fixation and permeabilisation may affect protein recovery. Even with optimised staining, fixation, and permeabilisation conditions, ASAP-seq exhibits a two-fold reduction in signal complexity for protein tags compared to CITE-seq [23]. As mentioned, epigenetic changes in cells are also marked by histone modification. scCUT&Tag-pro can simultaneously profile six histone modifications with 173 surface proteins [24]. As a method specialised in profiling histone modifications, scCUT&Tag-pro provides detailed insights into chromatin states and is useful when specific histone marks are of interest.

### 2.5. Trimodal Omics Assays

More recently, researchers have extended multi-omics profiling to enable the simultaneous analysis of three omics in a single cell (Figure 1B, sample 5). Building on the 10× Multiome and the optimised conditions for preserving surface protein integrity in ASAP-seq, DOGMA-seq was introduced to simultaneously profile the epigenome, transcriptome, and proteome [23]. NEAT-seq (Sequencing of Nuclear Protein Epitope Abundance, Chromatin Accessibility, and the Transcriptome in Single Cells) also profiles the epigenome, transcriptome, and proteome but focuses on intranuclear proteins [25]. Staining intranuclear proteins often results in high background signals due to nonspecific oligo-antibody staining in the nucleus. NEAT-seq uses *E. coli* single-stranded DNA binding protein (EcoSSB) bound oligo-conjugated antibodies to neutralise the charge, improving the signal-to-noise ratio. Notably, NEAT-seq uses antibody-linked hashtag oligos targeting ubiquitous surface proteins to label samples. This demonstrates its potential to simultaneously profile intranuclear and surface proteins in the same cells. However, NEAT-seq is less user-friendly due to the extra conjugation and optimisation steps required for EcoSSB-bound oligo-conjugated antibodies. In contrast, DOGMA-seq is designed for seamless integration with existing workflows, leveraging commercially available reagents.

### 2.6. The Emerging Spatial Multi-Omics Technologies

All the assays described above achieve single-cell resolution but lack spatial context. Recently, a new generation of multi-omics technologies has emerged, enabling the profiling of cells in their in situ context. However, these technologies vary significantly in resolution, and many do not provide single-cell resolution. Spatial assays can be broadly categorised into two approaches: spatial indexing transcriptomics and imaging-based protocols [26].

Spatial indexing transcriptomics assigns RNA molecules to specific locations using barcoded arrays, followed by NGS to spatially map gene expression within tissues. Examples include Spatial Transcriptomics (ST), which has been commercialised as Visium (10× Genomics) [27]. In contrast, imaging-based techniques use fluorescence microscopy to directly visualise proteins or RNA molecules in situ. Examples include Phenocycler Fusion (10× Genomics) for protein visualisation, as well as MERFISH (Vizgen) and Xenium (10× Genomics) for targeted transcript profiling [28,29,30,31,32,33,34,35,36,37]. These imaging-based methods typically provide higher-resolution data at the cellular or subcellular level but are limited by lower-plex panels.

Methods that combine spatial transcriptomics and proteomics often rely on sequential data collection from the same tissue sample (Figure 1B, sample 6). ). In GeoMx DSP (GeoMx Digital Spatial Profiler), surface proteins are first stained with antibodies conjugated to ultraviolet (UV)-cleavable oligonucleotide barcodes [38]. A similar workflow then targets RNA using UV-cleavable barcodes attached to RNA hybridisation probes. Next, UV light releases barcodes from selected regions, which are then collected and analysed to profile proteins and transcripts. GeoMx DSP enables whole-transcriptome profiling alongside the analysis of over 570 proteins. The resolution (10 µm) is limited by the area illuminated by UV light per cycle [38]. Visium can also be coupled with protein detection, offering a resolution of 55 µm [27]. In this approach, oligonucleotide-bound antibodies stain proteins before tissue permeabilisation releases RNA. The Visium platform captures both RNA and oligonucleotide barcodes via spatial barcodes on a slide, allowing spatially resolved protein and RNA expression analysis. Compared to the iterative UV-cleavage and sequencing process in GeoMx DSP, Visium increases throughput but reduces resolution. To address this limitation, computational methods such as Stereoscope and CellTrek have been developed to deconvolute low-resolution images using scRNA-seq data as a reference [39,40]. 

Emerging tools like 10× Genomics Xenium complement Visium by offering higher -resolution on a single-cell or subcellular level [29]. Xenium employs on-instrument biochemistry and decoding cycles to preserve protein epitopes, enabling sequential protein detection. Notably, all Visium, GeoMx DSP, and Xenium support fresh frozen (FF) and formalin-fixed paraffin-embedded (FFPE) samples but only GeoMX DSP and Visium officially support fixed frozen tissue [27,28,29,38]. Xenium achieves higher resolution; however, it relies on a targeted spatial transcriptomics approach rather than the unbiased WTS in Visium and GeoMx DSP. Currently, 10× Genomics offers an optimised 5000-gene panel covering a broad range of genes across multiple organs and biological systems. Although highly comprehensive and demonstrative of Xenium’s capability for high-plex profiling, researchers typically use a much smaller subset tailored to specific studies. For instance, a melanoma TME study would exclude genes specific to other organs. Careful panel design for both gene and protein targets are essential for the Xenium platform.

Beyond commercial platforms, several laboratory-developed methods provide more cost-effective but less automated alternatives for co-profiling the transcriptome and proteome. The Spatial PrOtein and Transcriptome Sequencing (SPOTS) method enables the co-profiling of 200–300 proteins in mouse and cancer tissues at cellular resolution [41]. Similarly, Spatial-CITE-seq combines DNA-barcoded antibodies with microfluidic barcoding to assign spatial coordinates to both RNA and protein data. Spatial-CITE-seq has been applied to tissues such as the mouse spleen (189 proteins) and human biopsies (273 proteins) [18].

Spatial assays that co-profile epigenome with transcriptome or genome with transcriptome are also available but are less commonly applied in cancer research. MERFISH and seqFISH, initially developed for high-sensitivity spatial transcripts profiling, have been adapted for genome-scale chromatin tracing in DNAseqFISH+ and DNA-MERFISH [42,43]. However, these methods have only been applied to cultured cells, their utility in complex tissue samples remaining unexplored.

Spatial-ATAC&RNA-seq and spatial CUT&Tag-RNA-seq integrate spatial epigenomics with transcriptomics using microfluidic barcoding strategies like DBiT-seq [44]. In these approaches, tissue cryosections are spatially barcoded, and sequencing reads are assigned to a two-dimensional grid of barcoded pixels. These methods have been applied to mouse and human brain tissues, providing insights into how epigenetic states influence cell identity and dynamics [44]. However, achieving single-cell or higher resolution remains challenging.

**Table 1:** Summary of single-cell multi-omics assays.

Assays	Omics	Resolution	Sequencing Depth & Sensitivity ^†^	Throughput	Platform	Sample Compatibility	Cost ^	Reference
G&T-seq	DNA, RNA	Single-cell	DNA: up to 33×/cell RNA: 400,000 reads/cell	100–200 cells/experiment	Plate-based	Fresh cells, frozen cells	-	[6]
SIDR	DNA, RNA	Single-cell	DNA: 0.13–0.79×/cell RNA: not reported	Tens to few hundred cells/experiment	Plate-based	Fresh cells, frozen cells	-	[7]
DNTR-seq	DNA, RNA	Single-cell	DNA: 1 million read pairs/cell RNA: not reported Sensitivity: Number of genes/cell comparable to samrtSeq2 with same cell type	Thousands of cells/experiment	Plate-based	Fresh cells, frozen cells	-	[8]
TARGET-seq	DNA, RNA	Single-cell	RNA: 2.93 million reads/cell DNA: targeted proofing of mutation Sensitivity: detect mutation in 98.4% of all Jurat cells	Up to 4500 cells/experiment	Plate-based	Fresh cells, cryopreserved cells	-	[10]
DR-seq	DNA, RNA	Single-cell	RNA: thousands reads/cell DNA: 0.6–2.5×/cell	Tens of cells/experiment	Plate-based	Fresh cells	-	[9]
Paired-seq	Epigenome, RNA	Single-cell	DNA: 1500–2500 reads/nuclei RNA: 1000–2000 reads/nuclei	Millions of cells/run	Ligation-based combinatorial indexing	Fresh cells, cryopreserved cells, frozen tissue	-	[11]
10× Multiome	Epigenome, RNA	Single-cell	ATAC: 25,000–50,000 read pairs/cell RNA: 20,000–50,000 read pairs/cell Sensitivity: R^2^ = 0.89 compared to scRNA-seq; R^2^ = 0.931 compared to scATAC-seq	5000–10,000 nucleus/run	Droplet-based	Fresh cells, fresh/frozen tissue	$$	[12]
CITE-seq	RNA, protein	Single-cell	RNA: 20,000–50,000 read pairs/cell Protein: ~5000 read pairs/cell	8000–10,000 cells/run	Droplet-based	Fresh cells, cryopreserved cells	$$	[15]
ECCITE-seq	RNA, protein (CRISPR perturbations)	Single-cell	RNA: 10,000–50,000 reads/cell Protein: not reported (lower than RNA) Sensitivity: detect low abundance sgRNA in 93–98% cells; 90% of the antibody-derived tags detected in most cells.	Up to 5500 cells/run	Droplet-based	Fresh cells, cryopreserved cells	-	[16]
inCITE-seq	RNA, (intracellular) protein	Single-cell	Sequencing depth: not reported Sensitivity: median of 2655 UMIs/cell; median of 1158 genes/cell	10,000–20,000 cells/run	Droplet-based	Fixed cells	-	[17]
RAID	RNA, protein	Single-cell	Sequencing depth: not reported Sensitivity: Detected 4000–10,000 UMIs/ cell; median of 5552 genes/cell	1000–2000 cells/run	Plate-based	Fixed cells	-	[19]
AbSeq	RNA, protein	Single-cell	RNA: ~20,000 reads/cells Protein: ~30,000 reads/cells	~10,000 cells/run (up to 12 pooled samples)	Microwell-based	Fresh cells, cryopreserved cells	$	[22]
ASAP-seq	Epigenome, protein	Single-cell	Not reported ^#^	>7000 cells cells/run	Droplet-based	Fixed cells	-	[23]
scCUT&Tag-pro	Epigenome, protein	Single-cell	Not reported	Up to 60,000 cells/experiment	Droplet-based	Fresh cells	-	[24]
NEAT-seq	Epigenome, RNA, Protein	Single-cell	ATAC and RNA: 35,000–300,000 read pairs/cell Protein: 5000–7000 read pairs/cell Sensitivity: 13,500–16,900 RNA UMIs/cell; 4799–5100 genes/cell; 26,700 ATAC fragments/cell	1000–6000 cells/run	Droplet-based	Fixed cells	-	[25]
DOGMA-seq	Epigenome, RNA, Protein	Single-cell	Not reported *	>7000 cells cells/run	Droplet-based	Fixed cells	-	[23]
GeoMx DSP	RNA, protein	Subcellular	RNA: 30–100 read pairs/µm^2^, Protein: ~2 read pairs/ µm^2^ Sensitivity: detect as low as 2 copies per cell across 300 cells; some antibody approach single-cell detection.	8 slides/day (up to 12 region of interest/slide)	Spatial Indexing transcriptomics	FFPE, fresh frozen, fixed frozen	$$	[38]
Visium CytAssist Spatial Gene and Protein Expression	RNA, protein	55 µm	RNA: 20,000–50,000 read pairs/spot Protein: ~5000 read pairs/spot Sensitivity: Unique probe chemistry enables capture of lowly expressed RNA.	2 slides/run (5000 spots per slide)	Spatial Indexing transcriptomics	FFPE, fresh frozen, fixed frozen	$$	[27]
Xenium (with protein detection)	RNA, protein	Subcellular	Sequencing depth: Not applicable for image-based assay Sensitivity: Highly sensitive for RNA with probe hybridisation (outperform Visium)	2 slides/run	Imaging-based	FFPE, fresh frozen	$$$	[29]
SPOTS	RNA, protein	55 µm (Visium)	RNA: 50,000 reads/spot Protein: 8500 reads/spot	2 slides/run (5000 spots per slide)	Spatial Indexing transcriptomics	Fresh frozen	-	[41]
Spatial-CITE-seq	RNA, protein		Protein: ~885 protein UMIs/25 μm pixel RNA: ~1972 RNA UMIs/25 μm pixel	~2500–6400 single cells/run	microfluidic deterministic barcoding	Fresh frozen	-	[18]
DNA-MERFISH	DNA, RNA	~3 Mb/per locus ☨	Sequencing depth: Not applicable for imag-based assay	~100–500 cells/run	Imaging-based	Fixed cultured cells	-	[43]
DNAseqFISH+	DNA, RNA	Subcellular	Sequencing depth: Not applicable for image-based assay	~hundreds cells/experiment	Imaging-based	Fixed cultured cells	-	[42]
Spatial-ATAC&RNA-seq	Epigenome, RNA	20–50 μm per pixel	ATAC: ~14,284 unique fragments/pixel, RNA: ~1073 genes & 2358 UMIs/pixel	~10,000–250,000/per run	Microfluidic deterministic barcoding	Fresh frozen	-	[44]
spatial CUT&Tag-RNA-seq	Epigenome, RNA	20 μm per pixel	Histone: ~10,000 unique histone fragments/pixel (H3K27me3/H3K27ac) RNA: 1329–2011 genes/pixel	~10,000–30,000/per run	Microfluidic deterministic barcoding	Fresh frozen	-	[44]

† Sensitivity(genes detected/cell) may vary due to sample characteristic, sequencing depth, and panel design. ^ Cost for assays that are not commercialised is not compared. Generally, plate- or microwell-based platform can be less expensive for lower throughput experiment. Droplet-based methods become cheaper per cell at higher throughput. $ (thousands/run), $$ (thousands to ten thousand/run) , $$$ (ten thousand/run) refer to relative pricing comparison of each assay. The actual price depends on panel design, and capture area (for spatial assays). # While no raw reads per cell are reported, transcription start site enrichment and fragment complexity are comparable to 10× Multiome. ADT complexity is 1.7–2 fold lower than CITE-seq. * Number of genes per cell is comparable to 10× Multiome after required permeabilisation and fixation step. Transcription start site enrichment and fragment complexity are comparable with 10× Multiome. ADT complexity is 1.7–2 fold lower than CITE-seq. ☨: Measures spatial positions of chromatin domains that are ~3 Mb (Genomic Resolution) apart along the genome.

## 3. Computational Integration of Multimodal Omics

With the advancement of multi-omics assays, large amounts of data are generated, making data integration and analysis essential for extracting meaningful insights. Depending on the biological objectives and available data types, different integration tools are used to enhance downstream analysis. A comprehensive summary of available tools for various integration strategies can be found in other reviews [45,46]. Here, we highlight widely applied tools, focusing on their roles in addressing biological and clinical questions in cancer research. Briefly, data integration should incorporate horizontal integration for cross-sample comparisons, vertical integration for linking molecular layers, and mosaic integration for holistic, system-level insights.

### 3.1. Horizontal Integration

Horizontal integration combines omics datasets from the same molecular layer but from different sources, conditions, or samples. Horizontal integration is essentially formulated as a batch correction problem, aiming to remove technical effects across experiments while preserving genuine biological variation within and between datasets. Several horizontal integration tools have been developed for single-cell datasets, including Seurat, Harmony, LIGER, and scVI [47,48,49,50]. 

Canonical Correlation Analysis (CCA) integration is an MNN-based (mutual nearest neighbour) method available in the Seurat package [47]. In the original MNN method, a pair of cells—one from each batch—are considered MNNs if they are the closest neighbours to each other when compared across all cells in the opposite batch. Each MNN pair is assumed to represent the same cell type. The difference between their expression profiles, known as the batch vector, is used for batch correction. However, MNN assumes that batch effects are smaller than biological variation between cell types. If they are of a similar magnitude, this assumption becomes problematic. CCA integration in Seurat enhances the process by applying CCA for dimensional reduction before identifying MNNs. Additionally, each MNN pair is scored based on whether similar pairs are consistently identified between the same two clusters. In CCA integration, both the batch vector and confidence scores are used for batch correction. A slightly modified method, reciprocal principal component analysis (RPCA) integration uses principal component analysis (PCA) for dimensional reduction [51]. RPCA is designed to avoid overcorrection, making it more suitable when many cells do not overlap across batches. RPCA integration is also a faster method and is useful when integrating large datasets or multiple datasets generated by the same platform.

Harmony follows a different approach by first applying PCA for dimensional reduction [48]. It then employs a soft k-means clustering algorithm, where cells can belong to multiple clusters. A correction vector is iteratively computed to adjust for batch effects until cluster assignments stabilise. 

Beyond these widely used approaches, other methods offer alternative strategies for dataset alignment. Scanorama “stiches” multiple batches into a panorama by identifying similar cells across datasets using an MNN-based approach [52]. Scanorama uses singular value decomposition (SVD) for dimensionality reduction, followed by fast nearest neighbour search, to improve batch integration efficiency. Another MNN-based approach, Conos, construct a joint graph that captures plausible inter-sample mappings across datasets [53]. Conos preserve more biological variability by avoiding direct modification of expression values. This makes Conos well-suited for highly heterogeneous datasets with many unshared cell types. 

LIGER uses integrative non-negative matrix factorization (iNMF), which decomposes datasets into shared and dataset-specific metagenes [49]. Each cell is then assigned a label based on its maximum factor loading, and a neighbourhood graph is constructed using only shared factors to connect cells with similar factor-loading patterns. scVI utilises deep neural networks to compress scRNA-seq data into a low-dimensional latent space while accounting for technical noise and batch effects through a probabilistic model [50]. A variant of scVI for scATAC-seq data, PeakVI, has also been developed [54]. 

A standardised benchmarking study comparing popular horizontal integration tools is available to guide tool selection [55]. This study evaluates batch mixing efficiency using the k-nearest neighbor batch-effect test (kBET), assesses both batch integration and cell type preservation with the Local Inverse Simpson’s Index (LISI), quantifies the separation of batch-corrected cells from their original cell type using the Average Silhouette Width (ASW), and measures clustering accuracy relative to known biological labels with the Adjusted Rand Index (ARI). The rank sum identifies Harmony, LIGER, and Seurat as the top three performers overall, demonstrating consistent performance across multiple datasets. However, each tool excels in different aspects, and one may be favoured over another depending on the dataset and research needs. For example, Harmony excels at batch mixing for large datasets, whereas Seurat is computationally efficient and particularly effective when datasets contain overlapping cell types. Scanorama is well-suited for integrating datasets with minimal shared cell populations, while LIGER is ideal for preserving dataset-specific variation while still enabling integration. Beyond accuracy and integration quality, computational efficiency is another important factor. Deep learning models like scVI and PeakVI require substantial computational resources, whereas more traditional approaches such as matrix factorization (LIGER) or MNN-based methods (Scanorama, Seurat CCA/RPCA, Conos) tend to be less resource-intensive. Ultimately, the choice of integration method should be based on dataset characteristics, including data type, dataset size, the presence of shared cell populations, and the nature of technical effects.

While not explicitly designed for single-cell multi-omics, horizontal integration is essential for joint clustering and comparisons in both mono-omics and multi-omics studies. In large-scale sequencing projects (e.g., scRNA-seq) where data come from multiple batches or platforms (e.g., 10× Genomics vs. Smart-seq2), batch correction is necessary. When datasets originate from different conditions, time points, tissues, or species, horizontal integration helps remove technical effects and uncover true biological variability.

In cancer research, horizontal integration facilitates joint analyses and enables meaningful biological comparisons. For example, Seurat was used to remove batch effects and compare treatment-naïve and neoadjuvant chemoimmunotherapy-treated stage IIIA non-small cell lung cancer (NSCLC) patients. This analysis identified that B-cell IgG subclasses—IgG1 and IgG3—play a critical role in the anti-tumour immune response [56]. In the same study, a comparison between responders and non-responders revealed that a synergistic increase in B cells and CD4^+^ T cells was associated with a positive therapeutic response [56]. Additionally, samples collected at different time points are often integrated into a unified dataset to track longitudinal changes. Trajectory analysis can then reveal how molecular states evolve. For instance, Zhang et al. demonstrated that resistant mantle cell lymphoma (MCL) exhibited a progressive increase in oxidative phosphorylation (OXPHOS), MYC signalling, mTORC1 activation, and G2/M checkpoint deregulation [57]. Simultaneously, immune evasion in resistant MCL was driven by progressive CD8^+^ T-cell depletion, metabolic reprogramming, and abnormal cell signalling (CXCR4-Galectin-1 interactions). Moreover, multiple patient cohorts from different clinical trials are sometimes pooled to identify shared molecular signatures associated with therapeutic efficacy or resistance [58,59]. 

Apart from batch correction, transfer learning of the same data types from one dataset to another is also used for denoising purposes. Examples include SAVER-X, which combines a deep autoencoder with Bayesian hierarchical model to extract transferable gene–gene relationships from large public datasets and apply them to new target datasets [60]. SAVER-X enhances cell-type identification, improves gene expression quantification, and preserves biological variability while reducing technical noise. Other horizontal integration uses features from the same data type as anchors to provide a multi-omics view. Examples include multimodal intersection analysis (MIA), which integrates scRNA-seq and ST datasets [61]. Specifically, sets of cell-type-specific and tissue-region-specific genes are first delineated, followed by hypergeometric test to determine whether their overlap is higher or lower than expected by chance. Consequently, the spatial distribution of cell populations can be inferred in higher resolution.

### 3.2. Vertical Integration

Vertical integration combines different data types (e.g., DNA, RNA, chromatin, and proteins) from the same cells to explore their interdependencies. This integrative approach processes paired measurements across multiple modalities, enabling joint analysis to capture hierarchical relationships between molecular layers and uncover complex gene regulatory mechanisms. Several vertical integration methods have been developed for single-cell datasets, including Seurat, MOFA+, totalVI (for CITE-seq), and MultiVI (for 10× Multiome) [62,63,64,65].

While paired measurements allow unambiguous coupling of different molecular profiles within a cell, effective integration is crucial for maximising the interpretability and biological insights from multiple modalities. Joint clustering and analysis face several key challenges. First, data types differ significantly in their quality and information content. In CITE-seq, for example, proteins typically have higher abundance than RNA due to their stability. Consequently, RNA data may become less informative when both protein and RNA measurements are available for a particular gene. To address the varying importance of different modalities, Seurat developed an unsupervised weighted nearest neighbour (WNN) model that learns the relative utility of each data type [62]. WNN generates an integrated representation of multimodal data for each cell, maximising insights from both datasets during clustering and downstream analysis. This approach can also be applied to 10× Multiome data, which combines RNA and chromatin accessibility measurements.

Another challenge in cross-modal analysis is the inherent variation in data distributions due to biological differences and diverse measurement techniques. For instance, RNA is typically profiled as a count matrix and normalised into continuous data. DNA mutations are usually represented as binary data indicating presence or absence. MOFA+ addresses this challenge by incorporating three different likelihood models: Gaussian distribution for continuous data, Bernoulli distribution for binary data, and Poisson distribution for count data [63,66]. This statistical framework enables the integration of various data types while detecting both shared and data-specific heterogeneity across modalities. Additionally, MOFA+ handles missing values through imputation using learned latent features. MOFA+ performs simultaneous horizontal and vertical integration in multi-omics studies involving multiple conditions. In contrast, Seurat performs horizontal and vertical integration sequentially [47,62]. 

The totalVI also accommodates sample-level categorical covariates (e.g., therapeutic conditions) for simultaneous horizontal and vertical integration and can handle missing values [64]. However, unlike MOFA+’s factor analysis approach, totalVI employs a deep generative model to learn joint cell representations for downstream analysis. The abstract latent spaces learned by totalVI can be challenging to interpret biologically and may require further processing. In contrast, MOFA+ links factors to biological pathways and variance decomposition, making it ideal for studying regulatory mechanisms. Nonetheless, the authors claimed that totalVI’s unique protein background removal capability provides more accurate differential expression analysis. Additionally, while MOFA+ and WNN are generalisable across data types, totalVI is specifically designed for protein and RNA (CITE-seq) integration. A similar architecture was used to develop MultiVI for integrating scRNA-seq with scATAC-seq data, with potential expansion to include protein as a third modality [65].

### 3.3. Mosaic Integration

Mosaic integration combines datasets of both the same and different types, often across samples, time points, or spatial contexts, to create a comprehensive and multidimensional biological picture.

Several algorithms originally developed for horizontal and vertical integration have demonstrated mosaic integration capabilities. These include LIGER and MOFA+, which focus on shared factors or modalities, and multiVI and totalVI, which utilise variational autoencoders [49,63,64,65,67]. All these methods support missing value imputation. Recent development efforts focus on mosaic integration tools, aiming to map multi-modal data from different conditions or time points into a unified framework for a holistic understanding of cancer. UINMF and MultiMap were the first methods to incorporate unshared features, demonstrating their importance and suggesting the possibility of using unshared features alone for data integration [68,69]. spaMosaic leverages graph neural networks (GNNs) to enhance connectivity between datasets, enabling seamless integration in spatial multi-omics contexts [70]. While spaMosaic’s spatial awareness is valuable for integrating tissue-contextual spatial datasets, spatial mosaic integration tools are still in early development. Most works focus on demonstrating data imputation accuracy rather than translational applications [71].

In contrast, paired measurements (e.g., 10× Multiome, CITE-seq) with vertical integration offer computational advantages by unambiguously assigning different modalities to single cells. However, the sequencing capacity requirements for each modality can limit the overall sample size. Resource constraints such as budget and hardware limitations may therefore lead research groups to favour mosaic integration pipelines over vertical integration approaches.

The choice of integration framework depends on the specific biological or clinical questions being investigated. Horizontal integration is used for cross-sample comparisons, while vertical integration maximises biological insights from linked molecular layers. Mosaic integration aims to provide holistic system-level insights. By applying sequential horizontal and vertical integration or using a mosaic framework, multi-omics data (e.g., RNA-seq, proteomics, and ATAC-seq) from patients with known treatment responses can be integrated to reveal molecular signatures and regulatory pathways driving clinical outcomes [20]. Moreover, optimal vertical integration can yield cell embeddings that outperform single-modality analyses by incorporating multiple information sources [62]. Beyond response group comparisons, linking genomic mutations to transcriptomic or protein changes in longitudinal samples or pseudo-time analyses enables reconstruction of disease progression trajectories [72]. For example, differentiation events observed at RNA or protein levels can be traced to upstream amplification or point mutation events. When comparing different response groups, this approach can reveal resistance mechanisms across multiple regulatory layers, potentially identifying biomarkers that can be detected earlier at the genomic level.

## 4. Application of Single-Cell Multi-Modal Omics in Understanding Cancer Heterogeneity to Enhance Therapeutic Innovations

Cancer patients treated with immunotherapies often develop innate or acquired resistances. While numerous studies aim to uncover resistance mechanisms to guide the development of next-generation immunotherapies, the diverse responses to anti-cancer immunotherapy—including patients failing to respond to multiple treatments—highlight that therapeutic resistance stems from multiple distinct mechanisms [3]. Here, we discuss how single-cell multi-omics technologies, which combine advanced laboratory measurement techniques with computational integration tools, help unravel this heterogeneous landscape of resistance.

### 4.1. Melanoma

Melanoma is the most aggressive form of skin cancer, with an increasing incidence in recent decades [73]. Despite advancements in immunotherapies, 50% of melanoma patients still succumb to the disease due to therapeutic resistance, with TME heterogeneity being a key driver of resistance [3]. Single-cell spatial multi-omics enables the simultaneous characterisation of transcriptomic and proteomic landscapes while preserving spatial context, providing deeper insights into resistance mechanisms and potential therapeutic targets.

In a study recently published, Quek et al. utilised CITE-seq and CODEX (Co-detection by indexing) to analyse RNA and protein expression in melanoma patients [74]. Using protein modalities from each dataset as common features, the CITE-seq and CODEX datasets from five melanoma patients were integrated using Seurat (i.e., vertical integration) and STvEA. STvEA enables integration of similar data types (e.g., protein expression) and facilitates RNA transcriptome mapping to CODEX images [75]. This integrated analysis revealed both “physical” and “functional” resistance mechanisms. In patients with innate resistance, researchers observed an “immune-striving” TME characterised by peri-tumour lymphoid aggregates with low T cell infiltration. While lymphoid aggregates alone did not distinguish between responsive and non-responsive tumours, the integrated analysis revealed higher expression of genes like *CD37*, *MS4A1*, *BLK*, *CD79A/B*, and *TNFRSF13C* in responsive tumours’ lymphoid aggregates. This detailed analysis required both CODEX imaging for visualising lymphoid aggregates and scRNA-seq for comprehensive transcriptome profiling. However, integrating data from different biopsies presents challenges due to tumour heterogeneity. CITE-seq and CODEX samples may not fully represent each other. For instance, while single-cell sequencing of one sample indicated a TME lacking T cells, only one CODEX image section showed this characteristic. Increased tumour region sampling could help address this limitation. Despite these limitations, computational integration offers advantages over some spatial transcriptomics methods, which may lack single-cell resolution (e.g., Visium) or be highly costly (e.g., Xenium).

In another study, Pozniak et al. applied scRNA-seq and spatial transcriptomics (Visium) to 27 paired pre- and on-treatment melanoma samples [76]. They identified a mesenchymal-like (MES) melanoma cell population in treatment-naïve samples that expanded during ICI treatment, contributing to resistance in non-responders. *TCF4* is highly expressed in MES cells and acts as a master regulator. Mechanistically, TCF4 suppresses melanocytic markers, antigen presentation, and IFN signalling pathways, thereby shielding MES cells from T cell-mediated killing. While scRNA-seq identified *TCF4*-regulated MES cells as a key resistant phenotype, it also served as a reference dataset for deconvoluting low-resolution Visium data. By mapping the scRNA-seq data on the spatial transcriptomic data using CellTrek, the authors demonstrated that MES cells localise in regions with low immune activity, aiding their survival despite immune pressure. This study provided insights into both cell-intrinsic factors (via scRNA-seq) and cell-extrinsic factors (via spatial transcriptomics) that influence melanoma’s response to therapy. Pozniak et al. also suggested that TCF4 could be a potential therapeutic target for overcoming resistance and that detecting MES cells in early on-treatment biopsies could serve as a predictive biomarker for ICI resistance.

Multi-omics studies can provide both spatial and single-cell resolution data within a single molecular layer, as demonstrated by Pozniak et al. work. Moreover, paired measurements of two molecular layers, combined with vertical integration, offer a more comprehensive representation of cell-intrinsic features. For instance, Lischetti et al. applied CITE-seq to advanced and primary melanoma biopsies [77]. With WNN integration (Seurat) of protein and RNA modalities, the analysis identified a CD56-abundant metastatic melanoma cell population that was absent in primary melanoma. This finding suggests a potential therapeutic target. Additionally, the authors explicitly compared results with and without the protein modality. The comparison demonstrated that the CD56^+^ cluster would be overlooked without CITE-seq because the transcript for CD56 (*NCAM1*) was barely detected at the RNA level. Other markers that were “protein-only” included CD70, CD71, CD81, CD107a, and CD274. The likely explanation is the high dropout rate in RNA detection and the inherently lower abundance and stability of transcripts compared to proteins.

### 4.2. Breast Cancer

Breast cancer is another highly heterogeneous disease, with molecular subtypes exhibiting distinct genetic, transcriptomic, and microenvironmental characteristics that influence disease progression and treatment response. Single-cell multi-omics approaches aim to provide a comprehensive framework to dissect this complexity, enabling a deeper understanding of tumour evolution, resistance mechanisms, and immunotherapy targets.

Zhu et al. used G&T-seq to profile the DNA (i.e., CNV, SNV) and RNA of samples from a triple-negative breast cancer (TNBC) patient [72]. Although no vertical integration tools were mentioned, the study unambiguously coupled CNV, SNV, and RNA data at the single-cell level, demonstrating that all cells from this TNBC patient were highly genomically and transcriptionally consistent. The clones identified using CNV data were also detected with SNV and RNA data. However, the overlap of genes between CNV and RNA clones was generally higher compared to SNV and RNA clones, suggesting that CNVs have a greater influence on shaping the transcriptome than point mutations. This level of insight into regulatory networks is not achievable with genome or transcriptome data alone. Furthermore, by combining phylogenetic analysis (for CNV and SNV) with pseudotime trajectory analysis (for RNA), the study identified three evolutionarily distinct clones and reconstructed a simplified history of disease progression. Amplification or point mutation events that may have contributed to differentiation events were also mapped.

Hormone receptor-positive, HER2-negative breast cancer (HR+/HER2-BC) generally has a more favourable prognosis but still exhibits unpredictable progression and recurrence due to heterogeneity in the TME. Single-cell multi-omics can reveal novel prognostic factors in this subtype. Joo et al. performed both scRNA-seq and SIDR-seq on cancerous epithelial cells from 14 HR+/HER2-BC patient samples [78]. Clustering of scRNA-seq and SIDR-WTS datasets independently identified four functional tumour subtypes. Instead of computationally integrating the two transcriptome datasets, subtype signatures from the scRNA-seq clusters were used to annotate the SIDR-WTS clusters. The study demonstrated that the two sample sets were representative of each other and that SIDR-seq alone provided reliable transcriptome profiling with sufficient sequencing depth. By performing uniform manifold approximation and projection (UMAP) to embed SIDR-WGS dataset into SIDR-WTS, they showed that CNV frequency was higher in the proliferating and secretory subtypes than in the migratory and dysfunctional subtypes. Moreover, patients with both a high migratory signature (transcriptome) and *EGFR* gains (CNV) had poorer disease-free survival than those with either factor alone. This suggested that hierarchical interactions between molecular layers underlie disease progression mechanisms. This HR+/HER2-BC study again highlights the importance of simultaneously profiling multiple omics layers to uncover resistance heterogeneity.

Integrating multiple omics technologies provides a comprehensive view of the TME and can identify cell populations that may be overlooked with single-omics approaches. Wu et al. conducted CITE-seq on four breast cancer samples and vertically integrated RNA and protein data using the WNN algorithm in Seurat [79]. Their analysis revealed that PD-L1 and PD-L2 were co-expressed across four distinct myeloid cell types: CXCL10^+^ macrophages, FABP5^+^ lipid-associated macrophages (LAM), APOE^+^ LAM, and LAMP3^+^ dendritic cells. This co-expression contributes to an immunosuppressive TME. Interestingly, while similar findings were observed using scRNA-seq alone, *PD-L1/2* expression in the two LAM subtypes was initially overlooked. This discrepancy between transcriptomic and proteomic data underscores the need for multi-omics approaches, especially when clinically significant cell types may be overlooked. Indeed, survival analysis indicated that FABP5^+^ LAM is associated with poorer patient outcomes.

Recognising the complexity of TME heterogeneity and the growing emphasis on studying cancer holistically, Wu et al. proposed a novel breast cancer classification approach [79]. By performing spatial transcriptomics (Visium) on six breast cancer samples (two ER+ and four TNBC) and deconvoluting using Stereoscope, they identified nine tumour clusters, termed “ecotypes”. Unlike the traditional PAM50 classification, which categorises breast cancers based solely on cancer cells gene expression (e.g., HER2-enriched, Luminal A/B), ecotypes incorporate the cellular composition of both tumour and non-tumour cells, along with their spatial organisation. This integrative approach improves breast cancer classification by capturing the complex interplay of cellular and spatial dynamics, potentially enabling more personalised and effective treatment strategies.

### 4.3. Other Cancer Types

The application of single-cell multi-omics extends beyond melanoma and breast cancer, providing insights into tumour heterogeneity, evolution, and therapeutic resistance across various malignancies (Table 2).

#### 4.3.1. Neuroblastoma

Olsen et al. performed DNTR-seq on single cells from neuroblastoma tumours, integrating genome and transcriptome data to resolve tumour heterogeneity and clonal evolution [80]. By leveraging both modalities, they identified aneuploid Schwann Cell Precursor-like (SCP-like) subclones as early tumour-initiating populations. These subclones have chromosome 17 gains and later transitioned into malignant adrenergic tumour cells. Clinically, this finding underscores the role of early aneuploid events in tumour initiation, suggesting potential biomarkers for early neuroblastoma detection and novel therapeutic targets to intercept tumour evolution before full malignancy.

#### 4.3.2. Osteosarcoma

Similarly, Nance et al. integrated snATAC-seq and snRNA-seq (10× Multiome, WNN) to map tumour heterogeneity and regulatory networks in osteosarcoma [81]. Their study demonstrated that chromatin remodelling plays a key role in osteosarcoma progression, highlighting potential epigenetic targets for therapy. By performing UMAP and clustering separately with ATAC-seq and RNA-seq data, they showed that the weighted combination of snRNA and snATAC data produced a WNN UMAP that allowed for the most distinct separation of cell types. Some cell populations were more closely grouped in either the ATAC or RNA UMAP alone, reflecting relationships in epigenetic and transcriptomic programming. This study demonstrates how WNN integration enhances cell-type representation and clustering, facilitating the identification of novel cell populations.

#### 4.3.3. Head and Neck Squamous Cell Carcinoma

Karjosukarso et al. applied RAID-seq to simultaneously profile RNA and (phosphorylated) proteins in drug-tolerant head and neck squamous cell carcinoma (HNSCC) samples [20]. Through this integrated approach, they identified 40 genes associated with drug tolerance, forming a transcriptional signature linked to patient outcomes. ITGA6 emerge as a predictive biomarker when phospho-proteomic data indicated its functional link to FAK activation.

#### 4.3.4. Non-Small Cell Lung Cancer

Beyond non-spatial single-cell multi-omics, spatial profiling approaches can enhance predictive biomarker discovery. The spatial distribution of therapeutic targets influences treatment responses, as seen with bispecific antibodies (bsAbs) like KN046, which simultaneously target CTLA-4 and PD-1. To investigate spatially predictive biomarkers, Song et al. utilised NanoString GeoMx DSP to analyse over 1800 transcripts and 44 proteins across specific tissue regions in NSCLC [82]. While this study did not achieve single-cell resolution, they identified 18 spatially resolved stromal features that better predicted bsAb immunotherapy responses than PD-L1 expression or tumour mutation burden.

#### 4.3.5. Pancreatic Cancer

Moncada et al. (2020) integrates scRNA-seq and ST to map the cellular architecture of pancreatic ductal adenocarcinoma [61]. The study links cell type-specific gene expression from scRNA-seq with spatially enriched genes from ST, enabling the inference of spatial distribution of distinct cancer and stromal cell populations. The integration revealed co-localisation of stress-response cancer cells with inflammatory fibroblasts, suggesting a tumour-supportive niche that may contribute to cancer progression. Additionally, the study identified spatially organised immune microenvironments, with M1 macrophages enriched in inflammatory regions and M2-like macrophages near ducts, providing insights into tumour immune evasion.

#### 4.3.6. Pre-Clinical Cancer Models

ECCITE-seq is less widely applied in translational research with patient-derived samples, likely due to inherent difficulties in performing CRISPR perturbations on fresh patient samples compared to cell lines. However, ECCITE-seq remains valuable for studying the downstream effects of gene knockdown. Papalexi et al. applied ECCITE-seq to multiple cell lines (THP-1, K562, KG-1, and U937), and perturbed genes potentially involved in PD-L1 regulation [83]. Their analysis identified KEAP1 (Kelch-like ECH-associated protein 1) as a negative regulator of NRF2 (Nuclear factor erythroid 2-related factor), an upstream activator of PD-L1. This finding suggests a potential therapeutic target to enhance anti-PD-L1 or anti-PD-1 therapy. Other studies have used ECCITE-seq on patient samples to profile RNA, protein, and T cell receptor clones without CRISPR perturbation, likely due to ethical and technical challenges associated with handling patient samples [84].

## 5. Clinical Consideration and Future Directions

Single-cell multi-omics technologies have rapidly emerged in recent years and are now being applied in translational cancer research using clinical samples. However, their integration into clinical workflows remains limited due to several challenges.

First, there are no standardised protocols for single-cell and spatial multi-omics technologies. Variability in sample preparation, sequencing depth, and data processing introduces inconsistencies across studies and institutions. Establishing standardised workflows and international guidelines is critical for ensuring reproducibility and comparability across clinical datasets. The Human Cell Atlas initiative, for example, aims to develop standardised protocols for scRNA-seq, serving as a model for harmonising multi-omics assays. A practical guide to scRNA-seq for biomedical research and clinical applications is already available, and a similar framework is needed for multi-omics assays [85].

Second, the complexity of multi-omics datasets requires advanced computational approaches to extract clinically meaningful insights, which generally require bioinformatics expertise for preprocessing, integration, and interpretation. However, healthcare professionals such as clinicians often lack the necessary expertise to interpret multi-omics and spatial data. To bridge this gap, user-friendly artificial intelligence (AI)-driven bioinformatics platforms could be developed to automate data integration, visualisation, and analysis. This would enable clinicians to focus on clinical decision making without needing deep computational expertise. A more traditional approach involves the creation of pre-validated, clinically actionable biomarker panels to facilitate decision-making. As discussed earlier, several multi-omics predictive markers have been identified, but multi-centre validation studies are needed to confirm their predictive and prognostic value across diverse patient populations [79,82].

Third, the high cost associated with reagents, high-throughput sequencing, and computational infrastructure poses a significant barrier to large-scale clinical implementation. Encouraging industry partnerships to develop cost-effective reagents and platforms could help reduce financial burdens. Additionally, as reliable predictive and diagnostic markers are identified, clinical applications will shift away from high-plex panels, WTS, and WGS, which are primarily used in research. For instance, Mission Bio Tapestri supports DNA and protein profiling in hematologic malignancies, making it a more focused and efficient option in clinical application [86]. Another cost-reduction strategy is multiplexing, where multiple patient samples are pooled into a single sequencing run. Techniques such as combinatorial indexing and cell surface protein hashing increase sequencing throughput and significantly lower per-sample costs [87]. Integrating such targeted and multiplexed strategies could enhance the financial feasibility of multi-omics technologies for routine clinical use.

Another major challenge in integrating single-cell multi-omics into clinical workflows is the turnaround time for sequencing and data analysis. In oncology, the timely return of results is critical for real-time decision-making, particularly in cases where rapid therapeutic intervention is needed. As previously discussed, AI-powered bioinformatic tools could bridge the gap between clinicians and complex datasets. This should also facilitate the data analysis process. To further reduce turnaround time, computational efficiency should be enhanced. Distributed computing could enable multiple institutions or cloud-based AI nodes to analyse patient data simultaneously. This would split computational loads, significantly reduce bioinformatics processing times and ensure that multi-omics insights reach clinicians faster. Beyond the analysis of multiplexed multi-omics data, AI-driven predictive tools are emerging to infer high-plex imaging data from haematoxylin and eosin (H&E)-stained slides. Examples include GHIST, VirtualMultiplexer, and CAMIL, which can instantly analyse H&E-stained images to predict molecular markers, gene expression, and clinical outcomes [88,89,90]. This approach eliminates the need for separate single-cell omics profiling, thereby reducing processing time and costs.

## 6. Conclusions

Single-cell multi-omics technologies provide a holistic view of cancer by integrating multiple molecular layers, enabling a deeper understanding of tumour heterogeneity, evolution, and therapeutic resistance. By simultaneously profiling DNA, RNA, chromatin accessibility, and proteins at single-cell resolution, these approaches reveal intricate cellular interactions within the TME and identify mechanisms underlying immunotherapy resistance. Applications in melanoma, breast cancer, and other malignancies demonstrate the power of multi-omics in uncovering therapeutic targets.

Despite these advancements, challenges remain in standardising protocols, improving computational integration, and reducing costs and time to facilitate clinical adoption. The development of AI-driven bioinformatics tools and cost-effective targeted approaches will be critical for translating multi-omics insights into clinical practice. As technology continues to evolve, single-cell multi-omics is poised to play a crucial role in precision oncology, guiding more effective and personalised treatment strategies.

## Figures and Tables

**Table 2:** Recent studies utilised single-cell multi-omics strategies to uncover cancer resistance.

Cancer Type	Multi-Omics Assays	Integration Methods	Integration Frameworks	Finding	Reference
Melanoma	CITE-seq, CODEX	Seurat STvEA	Vertical integration, Horizontal integration	Identified *MITF*+*SPARCL1*+ and *CENPF*+ melanoma subclones emerging post-immunotherapy, promoting resistance through impaired antigen presentation and interaction with cancer-associated fibroblasts (CAFs). Lymphoid aggregates with high B cell signatures correlated with better survival.	[74]
Melanoma	Visium, scRNA-seq	Harmony	Horizontal integration	MES cells are localised in regions with low immune activity is selected for ICI treatment. *TCF4* is highly expressed in MES cells, acting as a master regulator by suppressing melanocytic markers, antigen presentation, and IFN signalling pathways	[76]
Melanoma	CITE-seq	Seurat	Vertical integration	CD56-abundant metastatic melanoma cell population that was absent in primary melanoma. Demonstrated that “Protein-only” markers are important.	[77]
Breast cancer (TNBC)	G&T-seq	Not mentioned	Not mentioned	Identified three evolutionarily distinct clones and reconstructed a simplified history of disease progression, mapping amplification or point mutation events that may have contributed to differentiation events.	[72]
Breast cancer (HR+/HER2-BC)	SIDR-seq, scRNA-seq	Harmony	Horizontal integration	Patients with both a high migratory signature (transcriptome) and *EGFR* gains (CNV) had poorer disease-free survival than those with either a migratory signature or EGFR gains alone.	[78]
Breast cancer	CITE-seq, Visium	Seurat	Vertical integration	PD-L1 and PD-L2 were co-expressed across four distinct myeloid cell types: CXCL10^+^ macrophages, FABP5^+^ lipid-associated macrophages (LAM), APOE^+^ LAM, and LAMP3^+^ dendritic cells	[79]
Pancreatic ductal adenocarcinoma	scRNA-seq, ST	MIA	Horizontal integration	Identified co-localization of stress-response cancer cells with inflammatory fibroblasts. Identified that M1 macrophages enriched in inflammatory regions and M2-like macrophages near ducts.	[61]
Neuroblastoma	DNTR-seq	Not mentioned	Not mentioned	Identified aneuploid Schwann Cell Precursor-like (SCP-like) subclones as early tumour-initiating populations, characterized by chromosome 17 gains and subsequent transition into malignant adrenergic tumour cells.	[80]
Osteosarcoma	10× Multiome	Seurat	Vertical integration, Horizontal integration	Chromatin remodelling plays a key role in osteosarcoma progression, highlighting potential epigenetic targets for therapy. Demonstrated that integration of ATAC and RNA-seq data provides a better presentation of cells and allows more distinct separation during clustering.	[81]
Head and neck squamous cell carcinoma	RAID-seq	Seurat	Horizontal integration, Vertical integration	ITGA6 was found highly expressed in a subset of drug-tolerant persister (DTP) cells. It is only predictive when FAK activation.	[20]
Non-small cell lung cancer	GeoMx DSP	Not mentioned	Not mentioned	Identified 18 spatially resolved stromal features that outperformed traditional biomarkers, such as PD-L1 expression and tumour mutation burden, in predicting responses to bsAb immunotherapy	[82]
Multiple cancer cell lines	ECCITE-seq	Seurat	Vertical integration, Horizontal integration	Identified KEAP1 (Kelch-like ECH-associated protein 1) as a negative regulator of NRF2 (Nuclear factor erythroid 2-related factor), which acts as an upstream activator of PD-L1, suggesting a potential therapeutic target to enhance anti-PD-L1 or anti-PD-1 therapy.	[83]
Cutaneous T cell lymphoma	ECCITE-seq	Seurat	Vertical integration, Horizontal integration	Findings suggest that the skin microenvironment in cutaneous T cell lymphoma drives a transcriptional response that fosters rapid malignant expansion, in contrast to the quiescent state observed in the blood, which may have implications for therapy effectiveness.	[84]
